# Editorial

**DOI:** 10.34763/jmotherandchild.20202403.edit.03_2020

**Published:** 2021-04-30

**Authors:** Anna Oblacińska

**Affiliations:** 1 Department of Child and Adolescent Health Institute of Mother and Child; Warsaw; Poland

## Dear Readers and Authors

The editors of the Journal of Mother and Child/Medycyna Wieku Rozwojowego invite you to read the third issue of our quarterly. The topics include perinatology, which includes breastfeeding as a public health issue, the genetic aspect of diseases and developmental disorders in children, new therapeutic and diagnostic methods in the rehabilitation of children with congenital diseases and developmental disorders, and an interesting case report and clinical report.

We start reading with a review article in the field of perinatal medicine, which takes up an important topic, i.e. the search for measurable biochemical parameters that can help in the active management of childbirth, the timely diagnosis of dystocia and the choice of the method of delivery (Pośpiech and Czajkowski). This study therefore also has implications for everyday obstetric practice.

A significant number of studies conducted so far indicate that the level of lactate in the amniotic fluid may be a new non-invasive diagnostic tool for the early prediction of prolonged labour and the need for immediate obstetric intervention. Lactate level measuring in amniotic fluid may simplify placing the patient in a group that will benefit from the oxytocin administration or in a group that will not benefit from a further prolongation of labour.

The second article describes a cross-sectional study that allowed to identify relationships between selected socio-demographic factors and the concentration of immunoglobulins in breast milk (Akhter, Aziz et al.). Despite the relatively small size of the studied group of mothers, the study is interesting due to the differences in the approach to breastfeeding in different populations. In the country of origin of the report, that is, Bangladesh, breastfeeding in many cases determines a child’s ability to survive in the face of economic hardship and the resulting malnutrition of children. For this reason, the composition of breast milk and the possibility of its analysis are of great importance. The higher concentration of IgA in comparison to breast milk in Europe and the authors’ explanation of this fact is interesting information.

The next papers present a new look at therapeutic and rehabilitation methods in genetically determined chronic diseases – depending on both the use of new devices (Walicka-Serzysko et al. – a new device for airway clearance in cystic fibrosis) and the assessment of the impact of the perception of a child with craniofacial defects on its rehabilitation (Milska et al.). Emotional expression and relationship with the child were discussed, as well as overgeneralisation of inferring traits concerning these children. The Overgeneralisation Effect Scale (OES) can be one of the instruments for detecting differences in the perception of the child to improve its rehabilitation outcomes. The work is supported by a rich photographic illustration.

The genetic aspect of diseases and developmental disorders in children is the content of the two articles. The first one is a short report on the first Polish patient with a novel de novo variant in the *MED13L* gene with severe intellectual disability, developmental delay and facial dysmorphia (Dawidziuk et al.). Remarkable findings, such as absolute lack of speech, strabismus, and self-destructing behaviour presented by the patient allow the further definition of the phenotypic spectrum of mental retardation and facial characteristics with or without the heart defect syndrome.

The second article is a very interesting case report of drug reaction with eosinophilia and systemic syndrome (DRESS), which is rare in childhood (Prylińska et al.). DRESS is a severe, idiosyncratic drug reaction characterised by a prolonged latency period. This is followed by various clinical symptoms, usually fever, rash, lymphadenopathy, eosinophilia, and a wide range of systemic symptoms ranging from mild to severe. The pathogenesis of the syndrome is not well understood and is expected to consist of at least a complex interaction between a deficiency of detoxification enzymes leading to the accumulation of drug metabolites, and the genetic link between HLA and possible virus-drug interaction related to viral reactivation. DRESS is more often diagnosed in adults, and therefore the presentation of a case of this disease in a child and an interesting authors’ discussion of the genetic and metabolic basis of the disease in the developmental age validate the value of this study.

Enteroviral infections in infants younger than 3 months old are common, underdiagnosed and can be life-threatening. A properly obtained patient history, repeatedly neglected, plays a key role in the diagnosis and treatment of these infections. The authors of the clinical report (Olchawa-Czech et al.) have shown that in patients with the most severe course of enterovirus infection, a thorough epidemiological history of the family is very important, and the suspicion of a viral infection and the provision of appropriate diagnostic materials can significantly speed up the diagnosis in newborn baby and help implement proper treatment.

We hope that the presented papers will meet your expectations and encourage you to read the next issues of our Journal.

